# PiNNwall: Heterogeneous Electrode Models from Integrating Machine Learning and Atomistic Simulation

**DOI:** 10.1021/acs.jctc.3c00359

**Published:** 2023-07-21

**Authors:** Thomas Dufils, Lisanne Knijff, Yunqi Shao, Chao Zhang

**Affiliations:** Department of Chemistry-Ångström Laboratory, Uppsala University, Lägerhyddsvägen 1, P. O. Box 538, 75121 Uppsala, Sweden

## Abstract

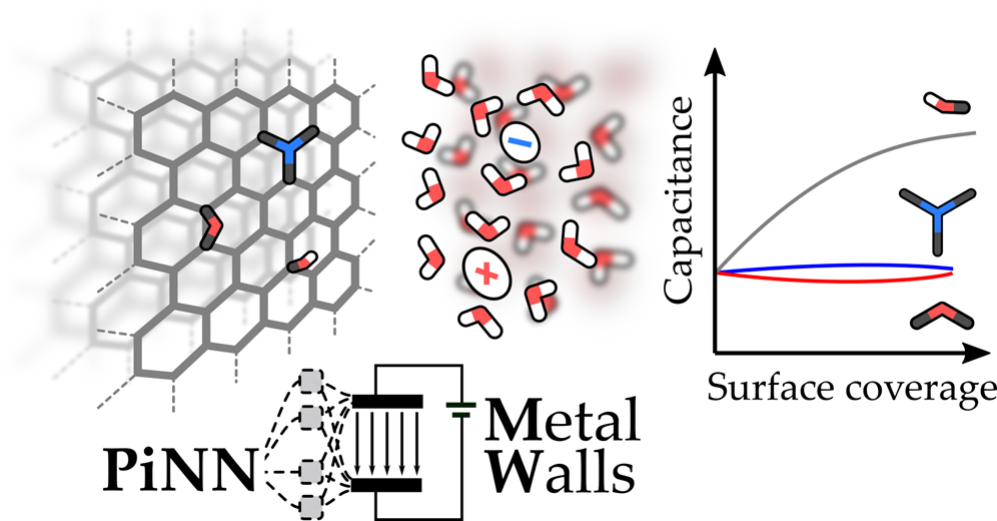

Electrochemical energy storage always involves the capacitive process. The prevailing electrode model used in the molecular simulation of polarizable electrode–electrolyte systems is the Siepmann–Sprik model developed for perfect metal electrodes. This model has been recently extended to study the metallicity in the electrode by including the Thomas–Fermi screening length. Nevertheless, a further extension to heterogeneous electrode models requires introducing chemical specificity, which does not have any analytical recipes. Here, we address this challenge by integrating the atomistic machine learning code (PiNN) for generating the base charge and response kernel and the classical molecular dynamics code (MetalWalls) dedicated to the modeling of electrochemical systems, and this leads to the development of the PiNNwall interface. Apart from the cases of chemically doped graphene and graphene oxide electrodes as shown in this study, the PiNNwall interface also allows us to probe polarized oxide surfaces in which both the proton charge and the electronic charge can coexist. Therefore, this work opens the door for modeling heterogeneous and complex electrode materials often found in energy storage systems.

## 1 Introduction

Electrochemical energy storage systems are indispensable components for building a sustainable and fossil-free society with infrastructures such as electric vehicles and energy grids. In particular, supercapacitors and batteries have attracted an ever-increasing attention in research going from materials chemistry to cell manufacturing. This is evinced by the 15 374 and 66 561 research articles published between 2020 and 2022 containing the keywords “supercapacitors” and “batteries,” respectively (source: the Web of Science), and highlighted by the 2019 Nobel Prize in Chemistry. On the other hand, to disentangle such complexity in these systems and to advance the field through fundamental insight, a physical approach is clearly needed.

Compared to battery systems, the capacitive charging process is the dominant one in supercapacitors. Indeed, electric double-layer capacitors (EDLCs) store energy from the electrostatic adsorption of ions on the electrode surface, which leads to a rapid charge–discharge cycle.^1^ In this case, the charge-transfer rate is vanishingly small, and the electrode can be considered as an ideally polarizable electrode.^2^ This means that chemical reactions and chemisorptions may be excluded from the setting;^3^ therefore, force field-based classical molecular dynamics (MD) is sufficient to simulate EDLCs.

The standard model for describing the charge distribution of polarizable electrodes is the Siepmann–Sprik model.^4^ It was improved by Reed and Madden^5^ to model perfect metal electrodes. Further improvements were done to account for the metallicity of the electrode material.^6^ This model has the advantage over other methods such as the image charge method^7^ to allow dealing with complex geometries, such as porous and disordered ones.^8^

**Figure 1:**
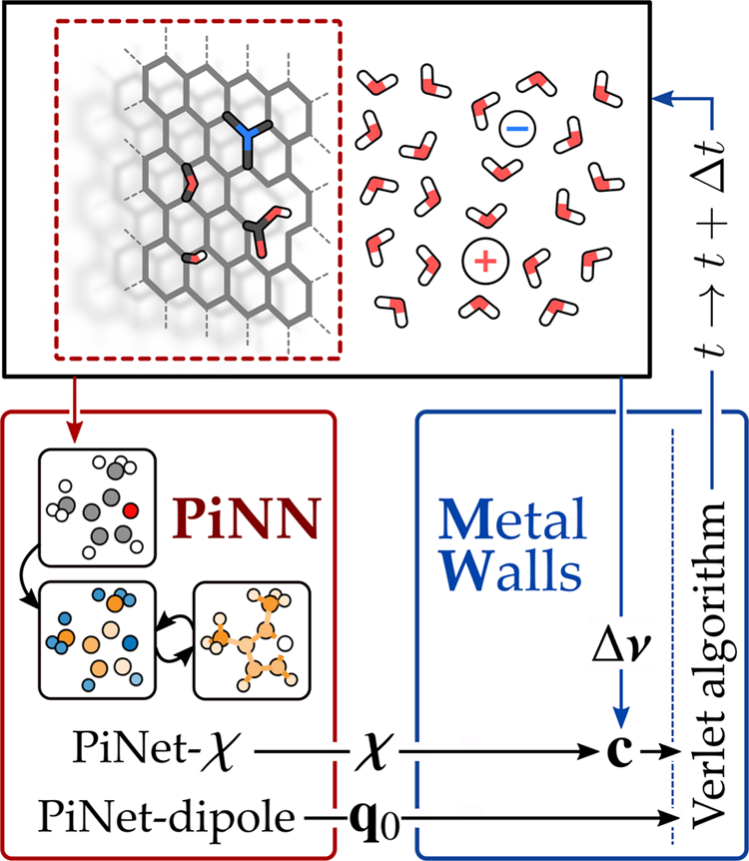
Flowchart of the PiNNwall interface. The electrode structure is passed from MetalWalls to PiNN, which computes the charge response kernel **χ** using PiNet-χ and the base charges of the electrode atoms *q*_0_ using PiNet-dipole. From the electrolyte configuration and the electrostatic boundary conditions, MetalWalls computes the potential on the electrode sites Δ***ν***. By combining χ and Δ***ν***, MetalWalls generates the response charges ***c*** at electrode sites, computes forces, and propagates the dynamics of the system using, for example, the Verlet algorithm.

Despite being successful for describing both the perfect metal (PM) electrode and the Thomas–Fermi (TF) electrode, the Siepmann–Sprik model does not naturally account for chemical heterogeneity.^9−14^ This is also true when it comes to the local effects of electrode geometry and atomic lattice disorder on metallicity. To account for the impact of the chemical heterogeneity of the electrode material on the response charge distribution, our approach here is to integrate machine learning (ML) and atomistic simulation with the PiNNwall interface, as shown in Figure 1. The purpose of this interface is to read the electrode structure from the classical MD code MetalWalls^15,16^ to compute the charge response kernel and the base charge with the atomistic ML code PiNN^17^ and then pass these info back to the MetalWalls for computing the response charge at electrode sites and propagating molecular dynamics simulations. By doing so, we can take advantage of both the efficient implementation of ML models in PiNN and the optimized computation of electrostatic interactions in MetalWalls.

In the following, we will first outline the computational methods used in this study including the theoretical formulation. This is followed by the implementation and the validation of the PiNNwall interface to make sure of its technical soundness. Then, the PiNNwall interface is applied to several cases of chemically doped graphene and graphene oxide where the chemical heterogeneity becomes important. In particular, we have showcased an example of graphene oxide terminated with deprotonated carboxylic groups where both the electronic charge and the proton charge are present. Finally, we close up with a discussion of future works.

## 2 Computational Methods

### 2.1 Siepmann–Sprik Model for a Polarizable Electrode

The basis of the Siepmann–Sprik model is to allow the electrode charges to fluctuate in response to the external potential. Each response charge of the electrode atoms follows a Gaussian distribution of magnitude *c*_*i*_ centered on the position of the electrode atom ***R***_*i*_
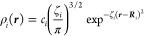
1where ζ_*i*_ is an adjustable parameter related to the Gaussian width.

The original model can be written as follows
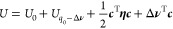
2where *U*_0_ corresponds to the energy of electrode atoms in the absence of an external potential (field), the term *U*_*q*_0_–Δ***ν***_ corresponds to the electrode–electrolyte interaction (so electrostatic interactions between the atomic charges of electrolyte atoms and the base charges ***q***_0_ of electrode atoms plus their van der Waals interactions), ***η*** is the hardness kernel, describing the interaction between response charges, and Δ***ν*** is the potential generated by the electrolyte at the electrode atom sites. It is worth noting that the formulation of the Siepmann–Sprik model shown here follows the linear response theory used in the chemical potential equalization method from York and Yang.^18^ This is different from other similar schemes,^14,19^ in which the atomic electronegativtiy were introduced to determine the base charge distribution *q*_0_. For historical developments on this topic and the subtle (yet important) difference in various schemes, we refer interested readers to our previous work^20^ and the atom-condensed Kohn–Sham DFT approximated to second order (ACKS2) paper^21^ for extensive discussion and references.

This energy is minimized with respect to the response charge ***c*** at each MD time step under the constraint of charge neutrality, which results in a linear relation between the response charge and the external potential as
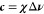
3where **χ** is the charge response kernel (CRK). It is related to the hardness kernel through^22^
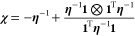
4where the second term of the right-hand side comes out from the charge neutrality constraint.

The finite-field extension in the case of a constant external field ***E***_0_ is straightforward, which leads to the solution of the response charge as
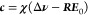
5It is worth noting that the external field ***E***_0_ equals to the Maxwell field ***E*** under periodic boundary conditions (PBCs).^23^

### 2.2 Response Charge Predictions from PiNet-χ

PiNet-χ^20^ is a graph convolution ML based on PiNet for predicting the linear response function CRK by regressing the molecular polarizability, as implemented in PiNN code.^17^

In this study, we used PiNet-χ which has been trained on the QM7b dataset^24^ to reproduce molecular polarizabilities computed from the density functional theory (DFT)^25^ with the B3LYP functional.^26,27^ Thus, it is suited to model electrode materials composed of the following elements: C, N, O, H, S, and Cl, which will be sufficient to study graphene (or graphite) and its derivatives, being amorphous graphene, nitrogen-doped graphene, or graphene oxides.

There are four different types of models provided by PiNet-χ, namely, the electronegativity equalization method (EEM)-type,^28,29^ the Local-type,^20^ the EtaInv-type,^20^ and the ACKS2-type.^21^ In the following, the essence of each model is summarized, and more details can be found in ref (20).

In the EEM-type model, the hardness matrix **η** is approximated by **η**_e_. **η**_e_ contains environment-dependent on-site hardness parameters, as well as the Coulomb kernel due to electrostatic interactions. From this, **χ** can be computed according to eq 4.

In the Local-type model, the polarizability tensor is constructed as the sum of atomic contributions **α**_*i*_. Then, the atomic contributions **α**_*i*_ are constructed from atom-centered predictions **χ**_*i*_ in a way that ensures translational and permutational invariance and rotational covariance. **χ**_*i*_ can be seen as atomic contributions to the CRK and are used to construct **χ** in the end.

In the EtaInv-type model, **χ** is constructed by predicting directly the softness matrix **η**^–1^. Besides the nearsightedness character of **η**^–1^, this type of models are computational efficient since the need for a matrix inversion operation is bypassed.

Finally, in the ACKS2-type model, two quantities are predicted instead, namely, **χ**_s_ and **η**_e_. Here, **χ**_s_ is constructed as a matrix that is local and trainable using symmetrized pairwise interactions. **η**_*e*_ is done in the same way as in the EEM model. These two predicted quantities can then be combined to construct **χ** through the Dyson’s equation, as shown in ref (20).
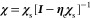
6

### 2.3 Base Charge Predictions from PiNet-Dipole

PiNet-dipole^30^ is a graph convolution ML based on PiNet as implemented in the PiNN code.^17^ The principle behind the PiNet-dipole model is to regress dipole moment/polarization data instead of atomic charge data, as the latter cannot be uniquely determined.

Here, a variant of PiNet-dipole trained on the QM7b dataset^24^ was used to be compatible with PiNet-χ. The model was trained using the following loss function
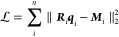
7where ***R***_*i*_ is a 3 × *N*_*i*_ matrix of the atomic coordinates of the configuration *i* for a molecular configuration containing *N*_*i*_ atoms, ***q**_i_* represents a column vector of the atomic charge, and ***M***_*i*_ is the corresponding dipole moment.

During the charge prediction phase, the base charge ***q***_0_ is obtained by
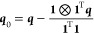
8This means that the total charge after charge prediction is evenly spread over all of the atoms in the system, resulting in a zero total charge in ***q***_0_.

In the case of protonated and deprotonated carboxyl groups, the total charge of ***q***_0_ of each carboxyl group is either +1 or −1. This constraint was implemented by adjusting the base charge of the carbon atom in the carboxyl groups.

Details of the validation and the implementation of base charges predicted from PiNet-dipole can be found in Section B of the Supporting Information.

### 2.4 Molecular Dynamics Simulations with MetalWalls

The MetalWalls code^15,16^ was used as the MD engine, which was built for simulating electrochemical systems with Siepmann–Sprik-type models. The box lengths in the different directions are *L*_*x*_ = 31.974 Å, *L*_*y*_ = 34.080 Å, and *L*_*z*_ = 70.124 Å. We use 3D PBCs, with Ewald summation used to compute electrostatic interactions with a real-space cutoff of 15.99 Å, the same cutoff being used for the Lennard-Jones interactions.

The electrode consists in 7 graphene layers with an interlayer spacing of 3.354 Å, resulting in 2912 carbon atoms, which leaves a 50 Å space along the *z* direction for the electrolyte. For each dopant type, we investigated, on top of the pristine case, two surface coverages: 10 and 20%. Only the graphene layers at the interface with the electrolyte are functionalized. In the case of nitrogen substitution, the atoms are placed randomly under the constraint that two nearest neighbor atoms cannot be substituted. For the doping with epoxy and hydroxyl groups, we used the rules for the amorphous graphene oxide model described in ref (31). Lennard-Jones parameters of electrode atoms were taken from the OPLS-AA force field^32^ with the use of the Lorentz–Berthelot mixing rules to compute the cross pair parameters with the electrolyte.

The simulation setup for the case of graphene oxide with the carboxyl termination is very similar to the case of the protonic double layer at metal oxide/electrolyte interfaces, as studied previously with finite-field DFTMD,^33,34^ in which two sides of an electrode take the same amount but opposite types of proton charge.

As for an electrolyte, we used an aqueous potassium chloride solution with a concentration of 1 mol/L, whose initial configuration has been generated with fftool^35^ and PACKMOL.^36^ This results in 1901 water molecules and 35 ion pairs. Water was modeled with the TIP3P model,^37^ and the ion models of aqueous K^+^ and Cl^–^ were taken from ref (38), which have been validated for high salt concentrations.^39^

The potential dependence is controlled through the finite-field methods adapted to the Siepmann–Sprik model,^40^ using *E* field values corresponding to potential differences across the simulation cell of 0 and 2 V. Each simulation consists in an equilibration run of 2 ns, followed by a production run of 10 ns. We used a time step of 2 fs in the NVT (constant number of particles, constant volume, and constant temperature) ensemble using the Nosé–Hoover thermostat^41,42^ with a relaxation time of 0.1 ps and a temperature of 300 K.

## 3 Implementation and Validations of PiNNwall

### 3.1 Passing the Charge Response Kernel from PiNN to MetalWalls

**Figure 2:**
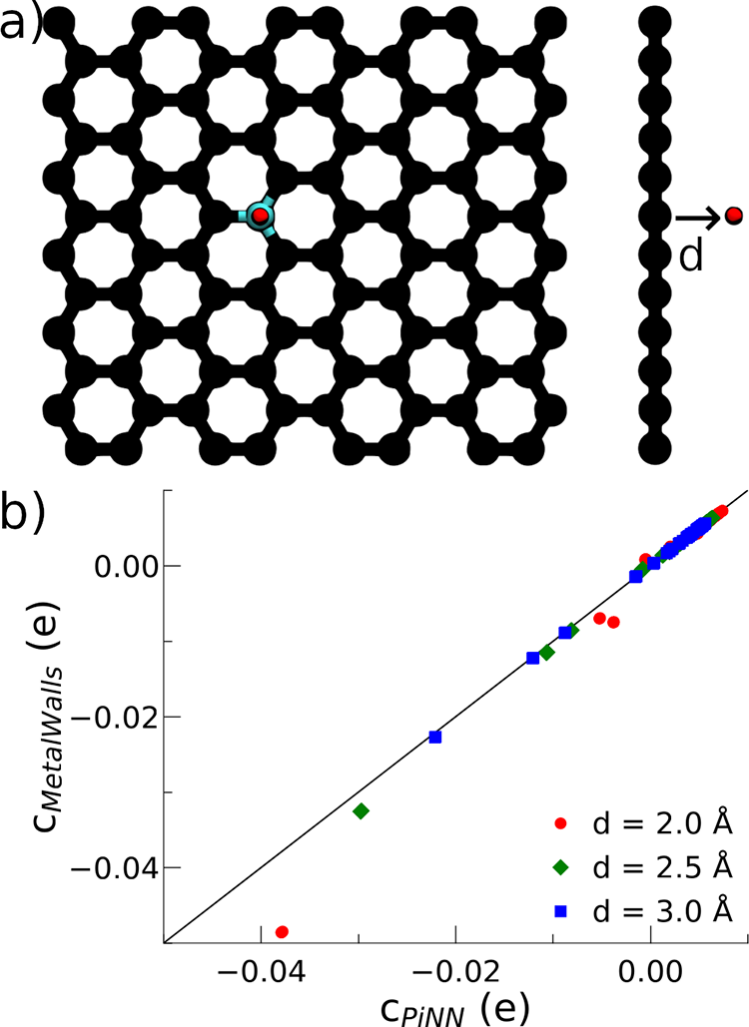
Passing the charge response kernel. (a) Nitrogen-doped graphene layer with 3D PBCs. A unit test charge is put at a distance *d* away from the surface on top of the defect. (b) Response charges predicted by MetalWalls via the PiNNwall interface against the prediction from PiNN using the same kernel PiNet-χ (EEM).

To test that the CRK **χ** is properly passed to MetalWalls through the PiNNwall interface, we consider the system described on Figure 2a: a nitrogen-doped graphene layer with 3D PBCs. A unit test charge is placed away from the surface on top of the defect with a distance *d*. Then, the response charges were computed with the same EEM-type models using PiNet-χ and MetalWalls. Results are shown in Figure 2b. One can see that the response charges agree very well with each other when the test charge is further away from the surface and only atoms that are second neighbor to the defect and beyond are considered. This indicates that the CRK is indeed successfully passed from PiNet-χ to MetalWalls via the PiNNwall interface. The discrepancies in other cases actually come from how the Ewald summation for computing the electrostatic potential due to the test charge was implemented. In PiNN, the electrode–test charge interaction was computed as a point charge–point charge interaction; in MetalWalls, the electrode–test charge interaction was computed as a Gaussian charge–point charge interaction instead. Nevertheless, such difference is immaterial and does not affect the passing of the CRK from PiNN to MetalWalls at all. Indeed, one can obtain a perfect agreement when choosing a smaller Gaussian width (Section A in the Supporting Information). It is worth noting that there is no need to choose the Gaussian widths when using the PiNNwall interface for practical applications (Section 4) as the Gaussian widths that were optimized in PiNet-χ (EEM) will be passed to MetalWalls for computing the electrostatic interactions. Therefore, there is no risk of double-counting of the screening effect and the implementation is self-consistent.

### 3.2 Forces and the Total Energy from the Charge Response Kernel

In contrast to the original Siepmann–Sprik model and its TF variant, the CRK instead of the hardness kernel **η** is the key quantity used in PiNet-χ. This means forces and the total energy in MetalWalls, that are formulated based on the hardness kernel, may not coincide with the CRK passed from PiNet-χ. Thus, we have to check the dependence on the hardness kernel of the quantities needed to run the MD and correct them if necessary.

**Figure 3:**
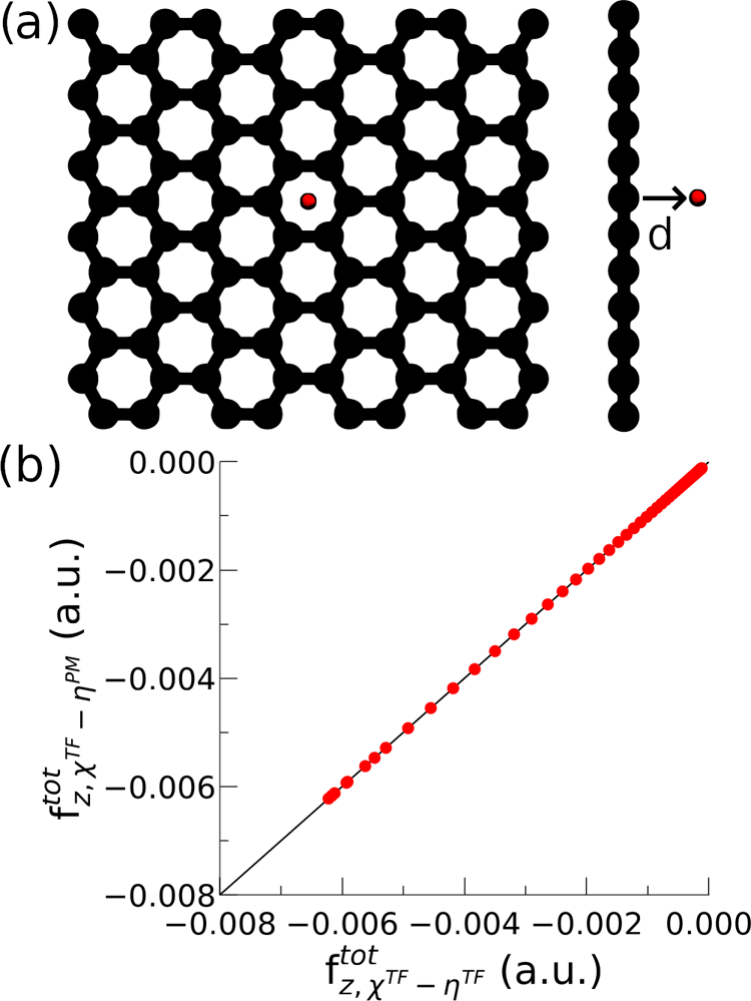
Forces from the charge response kernel. (a) A unit test charge is put on top of a graphene layer over the center of a six-membered ring, at a distance *d* of the layer, ranging from 0.5 to 5.5 Å. (b) Total contribution to the force acting on the test charge along the direction perpendicular to the surface. The subscript χ^TF^ – η^TF^ indicates both χ and η come from the Thomas–Fermi model. The subscript χ^TF^ – η^PM^ indicates χ comes from the Thomas–Fermi model, while η results from the perfect metal electrode.

To show whether these quantities depend on the hardness kernel or not, we use parameter sets of both PM and TF metals for constructing the hardness kernels **η** but only the parameter set of a TF metal for constructing the charge response kernel **χ**. Therefore, if the quantity in interest does not depend on **η**, then the results will lie perfectly along the diagonal line in the parity plot. As all of these tests were done with MetalWalls, we have used a system shown in Figure 3a: a unit test charge is put on top of a graphene layer over the center of a six-membered ring, at a distance *d* of the layer.

The forces caused by the interactions between the response charges and the electrolyte atoms at position ***r***_*i*_ are given by
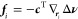
9

According to eq 3, the response charges depend only on the CRK. Since the external potential Δ***ν*** does not depend on the hardness kernel either, neither should the forces. Indeed, as shown in Figure 3b, the forces (acting along the perpendicular direction) are the same regardless of what **η** is used.

**Figure 4:**
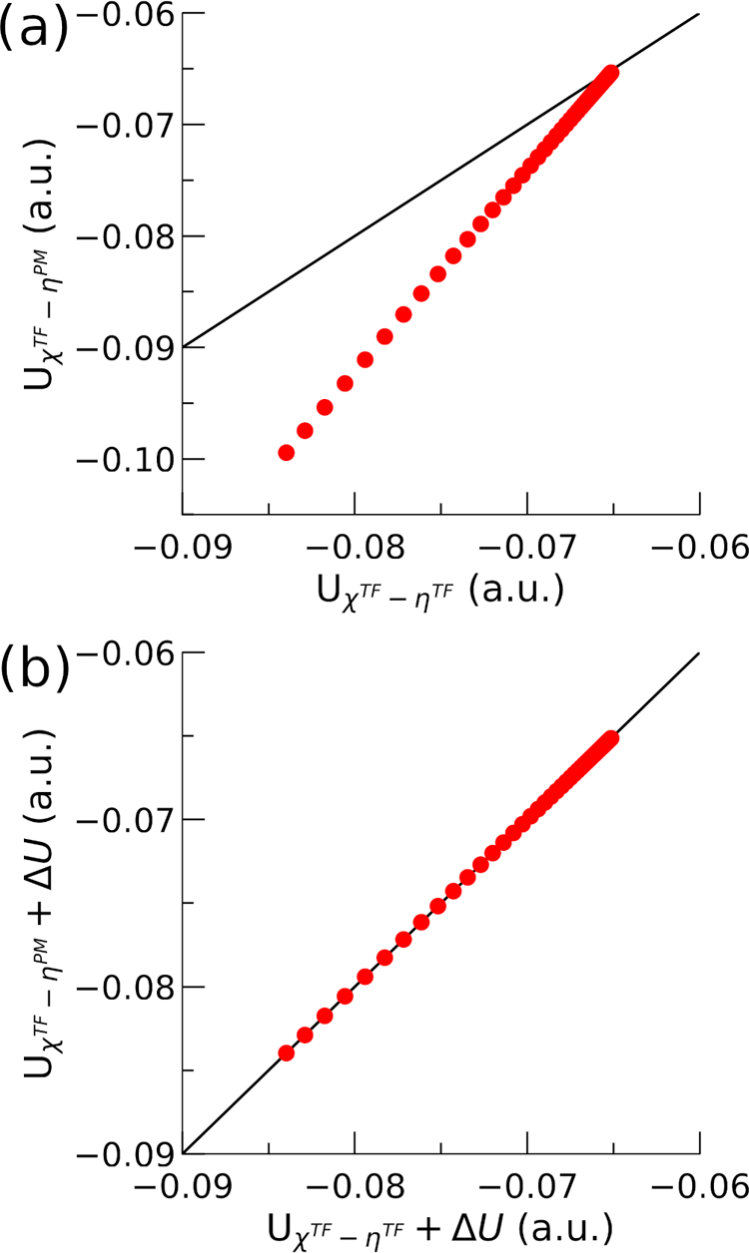
Hardness dependence of the total energy. Using the simulation setup of Figure 3a. (a) Without the correction term in eq 11, the total energy expression depends on both the hardness and the charge response kernel. (b) With the correction term in eq 11, the total energy depends only on the charge response kernel. The subscripts χ^TF^ – η^TF^ and χ^TF^ – η^PM^ follows the same convention used in Figure 3.

Next, we look at the total energy. According to eq 2, the total energy should depend on both the hardness and the charge response kernel. This is born out, as shown in Figure 4a. Therefore, one needs to resolve this discrepancy by rewriting the total energy expression in terms of Δ***ν*** and **χ** only.

As shown previously,^43^ the following equality holds under the variational condition
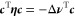
10

Thus, we can replace ***c***^T^**η*c*** with −Δ***ν***^T^***c*** and add a correction to the total energy as
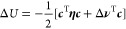
11

If the **η** – **χ** relation as defined by eq 4 is fulfilled, this term should be 0. A non-zero term arises when they are not self-consistent.

When applying this correction, the total energy does not depend anymore on the hardness used by the MD engine, as expected (Figure 4b). Thus, we now have everything checked to run MD properly with an ML-derived CRK via the PiNNwall interface.

### 3.3 Benchmarking on the Perfect Metal Electrode

**Figure 5:**
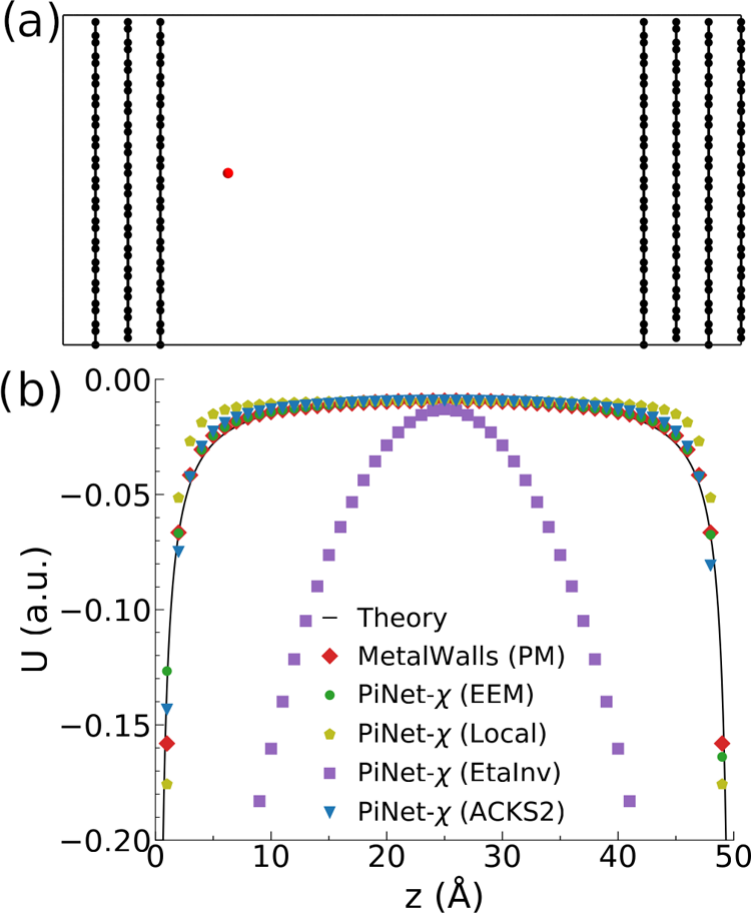
Electrostatic energy of a test charge between two sides of a graphite electrode. (a) The graphite electrode in vacuum under 3D PBCs is used as the model for representing a perfect metal electrode. (b) The total electrostatic energy of the system when moving the test charge between two sides of electrode. The solid line corresponds to the theoretical result , where *L* is the size of the vacuum slab and *z* the distance between the test charge and the electrode surface.

As a first test, a unit charge is put on top of the middle of a carbon ring of the interfacial plane and moved in the vacuum space between the two planes (Figure 5a). The total energy as a function of the charge position for the different models (MetalWalls, ACKS2, EEM, EtaInv, and Local) is displayed on Figure 5b along with the theoretical line. The ACKS2 and EEM are found more close to the theoretical line, which makes them the candidates for the next test. Note that MetalWalls (PM) throughout this work refers to simulations done with the default Gaussian width parameters as implemented in the code and originated from the work of Reed, Lanning, and Madden.^5^

**Figure 6:**
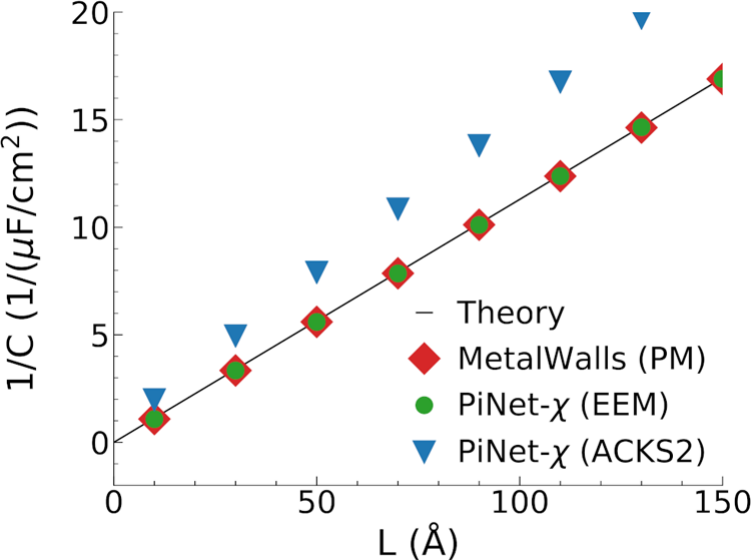
Inverse capacitance 1/*C* of an empty capacitor as a function of the vacuum slab size *L*. The system under consideration is the one shown in Figure 5a (without the test charge). The solid line corresponds to the theoretical result 1/*C* = *L*/ϵ_0_.

In the second test, we used the same graphite system as in Figure 5a and computed the corresponding capacitance by varying the size of the vacuum slab. When the graphite model behaves like a PM with the dielectric constant of infinity, the total capacitance will be only determined by the size of the vacuum. Its capacitance for the different models (MetalWalls, ACKS2, and EEM) as a function of the electrode separation is computed by applying a finite-field that leads to a potential bias of 2 V, and the results are displayed on Figure 6. In this case, the EEM kernel shows a metallic behavior and follows almost exactly the theoretical line, compared to ACKS2. The results of ACSK2 indicate that the electric field inside the graphite model is finite, which leads to a smaller polarization and a lower integral capacitance.

Based on these tests, we will employ the EEM kernel generated from PiNet-χ in the following case studies of chemically doped graphene and graphene oxide electrodes. In order to separate the effects of the local geometry and the chemical heterogeneity on polarizability, we will also employ a PiNet-χ model by considering all of the atoms as carbon atoms for the computation of the CRK, which is referred as PiNet-χ (EEM all C).

## 4 Application to Chemically Doped Graphene and Graphene Oxide Electrodes

### 4.1 Nitrogen-Doped Graphene Electrode

**Figure 7:**
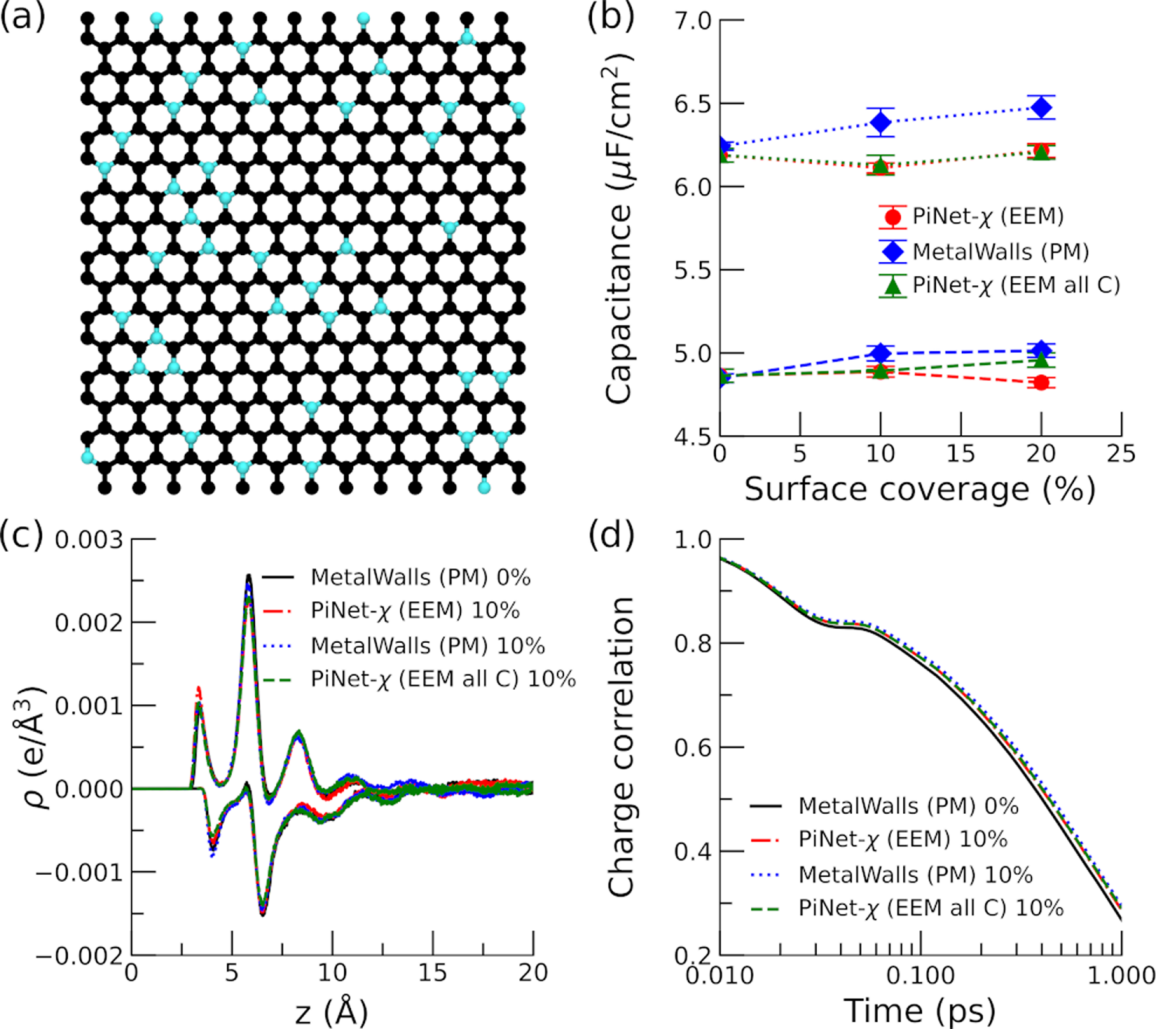
Nitrogen-doped graphene electrode. (a) Snapshot of the electrode surface with a 10% surface coverage (electrolyte solution is not shown for clarity). (b) Helmholtz capacitance for the positive and negative electrodes as a function of the surface coverage. Dashed lines correspond to the positive electrode while dotted lines correspond to the negative electrode. (c) Total charge density of ionic species as a function of the distance to the negative/positive electrode under an applied potential of 2 V. The distance is taken from the position of the carbon plane. (d) Time correlation function of the electrode charge under an applied potential of 0 V.

The simplest way to introduce chemical heterogeneity in the graphene layers is through the chemical doping, such as nitrogen, which shows a significant improvement on electrochemical activities.^44,45^ Due to its valence, nitrogen substitution does not induce an out-of-plane change in the layer structure itself (Figure 7a).

It is found that substituting carbon by nitrogen has a very limited impact on the Helmholtz capacitance (Figure 7b). This is also reflected in the charge density profile of ions next to the electrode as well as the dynamics of electrode charge (Figure 7c,d respectively).

We also notice that regardless of the model, the asymmetry in the Helmholtz capacitance between the positive and negative electrode remains, in which the capacitance of the negative electrode has a much higher capacitance at the same surface density. This is in accord with the observation that the cation distribution is more close to the electrode surface than that of anions.

### 4.2 Graphene Oxide Electrode with Epoxy Terminations

**Figure 8:**
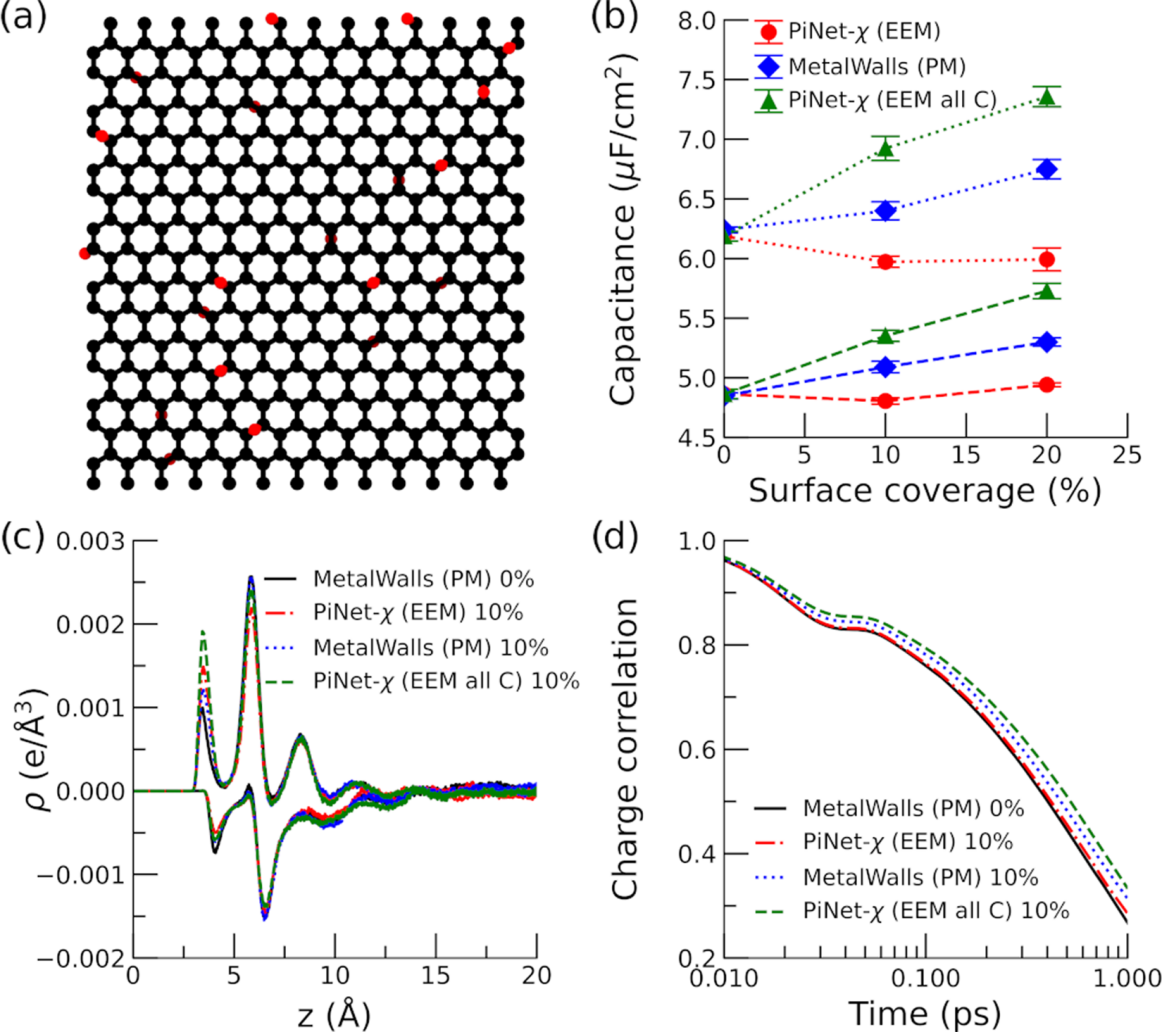
Graphene oxide electrode with epoxy terminations. (a) Snapshot of the electrode surface with a 10% surface coverage (electrolyte solution is not shown for clarity). (b) Helmholtz capacitance for the positive and negative electrodes as a function of the surface coverage. Dashed lines correspond to the positive electrode, while dotted lines correspond to the negative electrode. (c) Total charge density of ionic species as a function of the distance to the negative/positive electrode under an applied potential of 2 V. The distance is taken from the position of the carbon plane. (d) Time correlation function of the electrode charge under an applied potential of 0 V.

Epoxy, hydroxyl, and carboxylic acid functional groups are commonly found in the graphene oxide.^46^ In this section, we will look at how the Helmholtz capacitance will change upon introducing epoxy termination in the graphene oxide. This adds one layer of complexity as it also changes the roughness of the surface (Figure 8a).

In contrast to the case of the graphitic substitution as shown in the previous section, the doping with oxygen under the form of epoxy groups will modify the capacitance significantly (Figure 8b). Both PiNet-χ (EEM all C) and MetalWalls (PM) treat electrode atoms as carbon atoms regardless of element types, and yet PiNet-χ (EEM all C) shows a more rapid increment in the capacitance with the surface coverage compared to MetalWalls (PM). This highlights the fact that the CRK implemented in PiNet-χ does take into account the change in the “metallicity” due to the local geometry.

When comparing PiNet-χ (EEM all C) and PiNet-χ (EEM), the effect of chemical heterogeneity in the polarizability at atomic site comes into play. This in turn decreases the capacitance due to a smaller polarizability of oxygen and hydrogen atoms compared to that of carbon atoms. Therefore, the gain in the capacitance due to the surface roughness and the local geometry is canceled out by introducing the chemical heterogeneity.

As shown in Figure 8c,d, the charge density profiles of ions and the correlation function of the electrode charge do correlate with the observed capacitance. For instance, PiNet-χ (EEM all C), which has the highest capacitance, shows a strongest first peak of charge density for both positive and negative electrodes and the longest relaxation time. Nevertheless, this correlation is not perfect, in which the first peak height of charge density next to the negative electrode does no decrease in the same order as that in its capacitance. This suggests that the ion population in the second peak of charge density also contributes to the resulting capacitance.

### 4.3 Graphene Oxide Electrode with Hydroxyl Terminations

**Figure 9:**
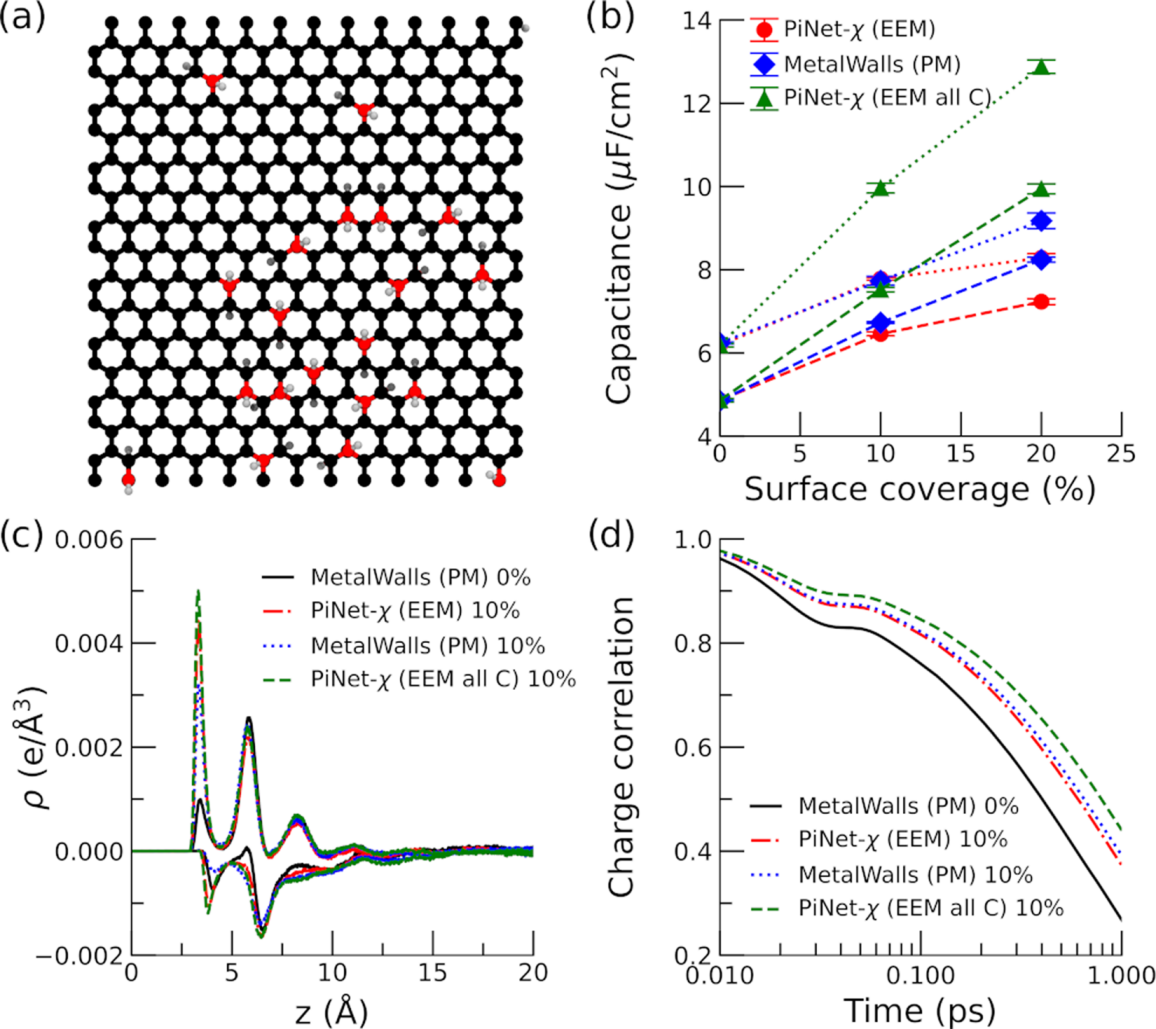
Graphene oxide electrode with hydroxyl terminations. (a) Snapshot of the electrode surface with a 10% surface coverage (electrolyte solution is not shown for clarity). (b) Helmholtz capacitance for the positive and negative electrodes as a function of the surface coverage. Dashed lines correspond to the positive electrode, while dotted lines correspond to the negative electrode. (c) Total charge density of ionic species as a function of the distance to the negative/positive electrode under an applied potential of 2 V. The distance is taken from the position of the carbon plane. (d) Time correlation function of the electrode charge under an applied potential of 0 V.

Next, we also looked into the case of the hydroxyl-terminated graphene oxide, as shown in Figure 9a.

In general, the trends for the capacitance (Figure 9b), the charge density profile of ions (Figure 9c), and the time correlation function of the electrode charge (Figure 9d) look similar to those observed in the case of the epoxy-terminated graphene oxide. Nevertheless, there are also considerable differences between the two cases. The capacitance obtained in the case of the hydroxyl-terminated graphene oxide is much higher than the epoxy case for the same surface coverage. Notably, the corresponding charge densities of ions at both positive and negative electrodes also have much higher intensities (Figure 9c). This suggests that by increasing the surface coverage of OH groups, the electrode surface becomes more hydrophilic and ion populations next to the electrode surface increase because of a more favorable solvation environment.

### 4.4 Graphene Oxide with Proton Charge

Examples in previous sections focus on the interplay between the geometrical effect on metallicity and the chemical heterogeneity in polarizability by comparing the perfect metal model in MetalWalls, PiNet-χ (EEM), and PiNet-χ (EEM all C). In this section, we will apply PiNet-χ (EEM) to probe the surface acid–base chemistry of electrode materials instead.

**Figure 10:**
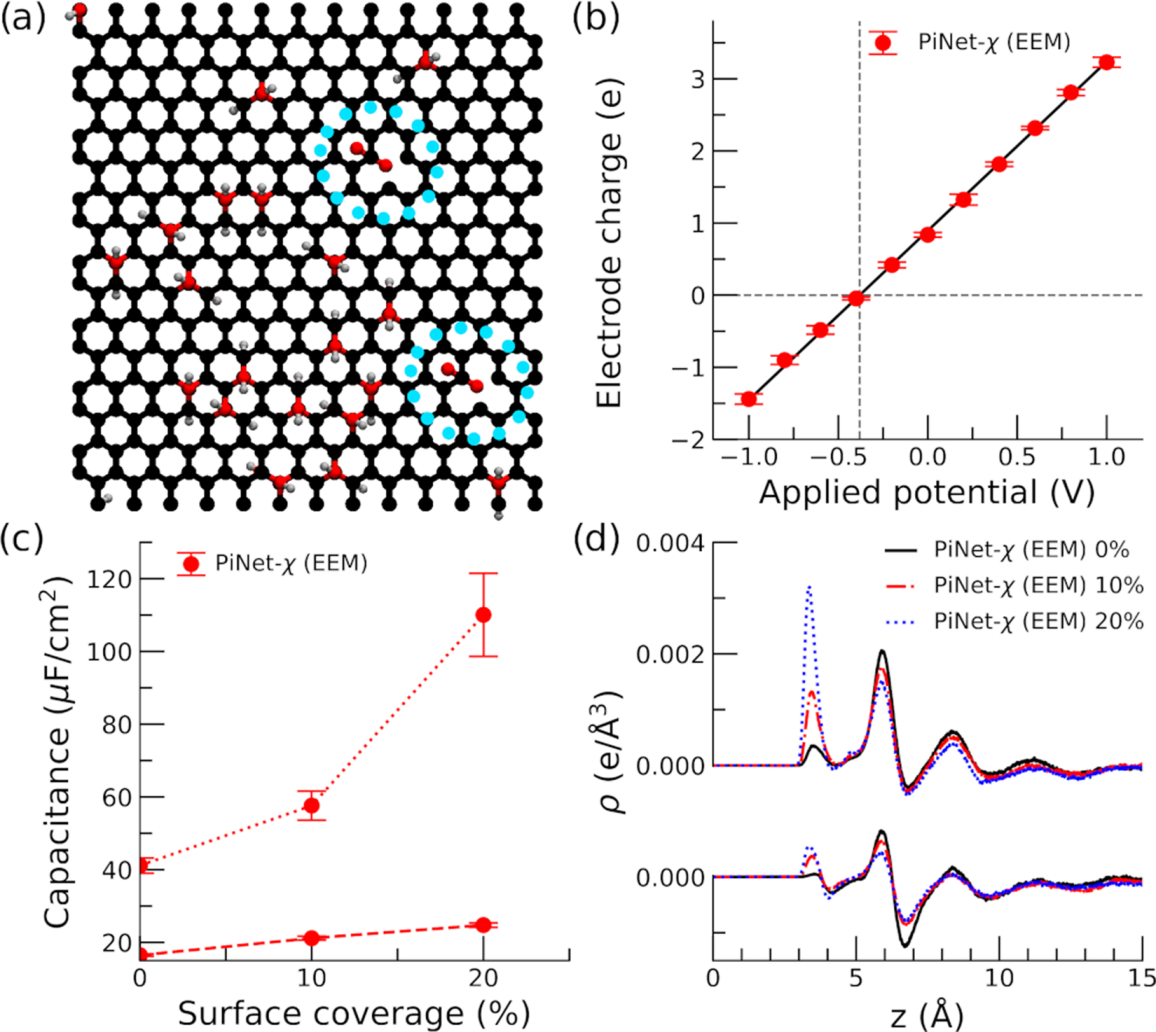
Graphene oxide with proton charge. (a) Snapshot of the carboxyl-terminated electrode surface with a 10% surface coverage of OH (electrolyte solution is not shown for clarity, and the location of deprotonated carboxyl groups are highlighted). (b) Electrode charge as a function of the applied potential. *V*_PZEC_ is identified when the electrode charge becomes zero. (c) Helmholtz capacitance for the positive and negative electrodes as a function of the surface coverage of OH. Dashed line corresponds to the positive electrode, while dotted line corresponds to the negative electrode. (d) Total charge density of ionic species as a function of the distance to the negative/positive electrode at the point of zero free charge (PZFC). The distance is taken from the position of the carbon plane.

In graphene oxide, both surface carboxylic and hydroxyl groups can undergo protonation/deprotonation depending on the solution pH. It has been reported that the *pK*_a_ is about 6.6 for the carboxylic group and 9.8 for the hydroxyl group in graphene oxide.^47^ This means that, at the neutral pH, the most relevant ionizable group in graphene oxide is the carboxylic group and the most probable acid–base reaction is the one shown in eq 12. Therefore, in this section, we will explore the PiNNwall interface for modeling the protonic double layer at the graphene oxide surface terminated with carboxylic groups (Figure 10a)
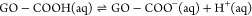
12
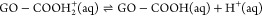
13

As shown in Figure 10b, by changing the applied potential, one can identify the point of zero free charge (PZFC) due to the electronic polarization. This “titration” procedure is similar to the one used before in modeling charged insulator/electrolyte interfaces for eliminating the finite-size effect.^48^ It is worth noting that the slope of Figure 10b yields a capacitance of value 4.7 μF/cm^2^, which is comparable to that of pristine graphene (see Figure 7b for the case of 0% surface coverage).

Once the PZFC is identified, the integral capacitance can be computed readily using the d*q*/d*V*_PZFC_ formula, in which *q* is the proton charge that we introduced through the protonation and deprotonation of carboxyl groups. The result of the computed Helmholtz capacitance due to the proton charge at the PZFC is shown in Figure 10c. What is surprising is that the resulting Helmholtz capacitance for the hydroxylated surface with deprotonated carboxyl groups can be as large as 100 μF/cm^2^. This is one order of magnitude higher compared to those found in pristine graphene but very similar in magnitude as those reported for metal oxide.^33,34^ Therefore, this finding provides a clue why the Helmholtz capacitance found in metal oxide is much higher than that found in the metal, as often seen in experiments.^49^

## 5 Conclusions and Outlook

In this work, we have integrated the atomistic ML code (PiNN) and the MD simulation code (MetalWalls) to model heterogeneous electrode surfaces. PiNN was used to generate the response kernel and the base charge from ML models PiNet-χ and PiNet-dipole, respectively. Then, this information was passed to the MetalWalls to carry out efficient computations of electrostatic interactions and to propagate the dynamics.

Through validation and verification, we have identified PiNet-χ (EEM) as the candidate for practical applications, which shows almost identical results for pure carbon electrodes compared to the original Siepmann–Sprik model. Thanks to the flexibility of PiNet-χ (EEM) for modeling any electrode materials composed of C, N, O, H, S, and Cl, we were able to study both chemically doped graphene electrode and graphene oxide with various terminations.

It is found that while the surface roughness and hydrophilicity can potentially increase the capacitance, these beneficial effects are attenuated by a smaller polarizability of elements (N, O, and H) involved in the chemical heterogeneity. On the other hand, we showed that the proton charge due to the surface acid–base chemistry at graphene oxide surfaces can lead to a significant increment in capacitance, which is comparable in magnitude (100 μF/cm^2^) to those reported in metal oxide-based systems.

Given that the capacitance is so different depending on whether the electronic or the protonic charge dominates, it would be interesting to study the transition between these two cases in future works, which can shed light on the electrochemical behavior of the “polarized oxide surfaces”.^50^ Reparameterizing PiNet-χ for transition-metal oxides or transition-metal dichalcogenides would allow us to investigate an even broader range of complex electrode materials in contact with both aqueous and nonaqueous electrolytes. In terms of the development of PiNNwall, future works can also be considered in the direction to pass the forces from PiNN to MetalWalls. In combination with the ML potential for modeling the electrode materials,^51−53^ this will enable us to study the electrode dynamics and its role in the electrochemical energy storage.

## Supporting Information Available

Further validation of passing the charge response kernel, details of the validation and the implementation of base charges predicted from PiNet-dipole, and ion distributions at the electrified interfaces (PDF)
