# Supplementary material for: PiNNwall: Heterogeneous Electrode Models from Integrating Machine Learning and Atomistic Simulation
Source: J Chem Theory Comput. 2023 Jul 21;19(15):5199–209. doi: 10.1021/acs.jctc.3c00359 (PMC10413855; doi:10.1021/acs.jctc.3c00359)
Supplement: Supplementary file 1 — ct3c00359_si_001.pdf [file ct3c00359_si_001.pdf]

PiNNwall: heterogeneous electrode models from integrating machine learning and atomistic simulation

Thomas Dufils[†], Lisanne Knijff[†], Yunqi Shao[†] Chao Zhang[†*]

[†]*Department of Chemistry - Ångström Laboratory, Uppsala University, Lägerhyddsvägen 1, BOX 538, 75121, Uppsala, Sweden*

chao.zhang@kemi.uu.se

Supporting Information

A Further validation of passing the charge response kernel

In the Figure 2 of the Main Text, we observed a difference in the response charge predictions of PiNN and MetalWalls using the same CRK, that we attributed to the use of point charge-point charge electrostatic interaction with the test charge for the former and Gaussian charge-point charge interaction for the later. Here we show a validation of this hypothesis by using the same setup as in the Figure 2 of the Main Text but with a Gaussian width of 0.2 Å instead. Results are shown on Figure S1. Indeed, one can see that the difference in response charges predicted by PiNN and Metawalls become negligible for all cases by decreasing the Gaussian width.

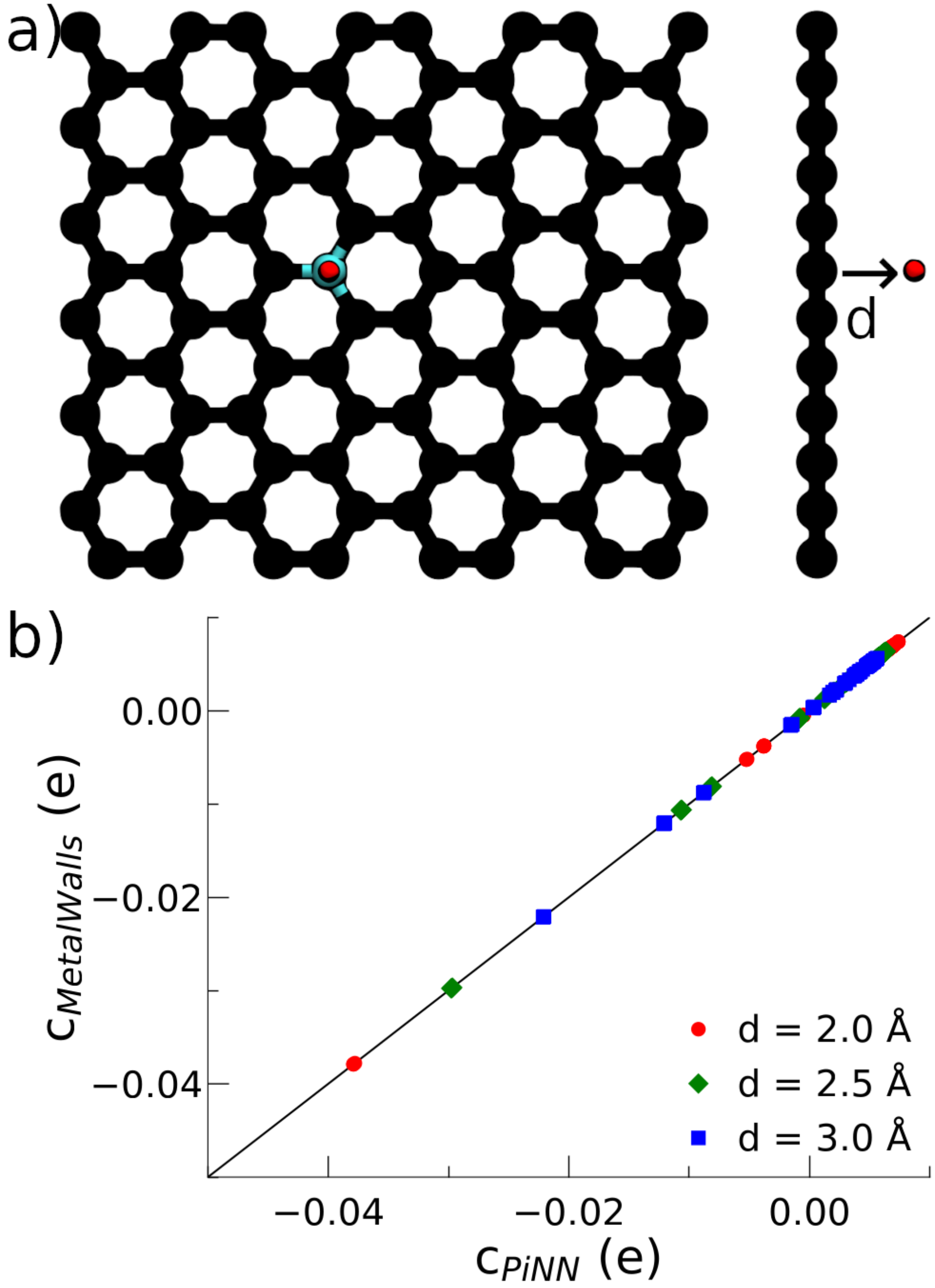

Figure S1: **Passing the charge response kernel**. Response charges predicted by MetalWalls via the PiNNwall interface against the prediction from PiNN using the same kernel PiNet-χ (EEM).

B Validation and implementation of base charges predicted from PiNet-dipole

To validate the charges predicted from the PiNet-dipole model, molecular analogues of the target structures were used for each of the functionalized graphene models. To validate that the charges predicted from PiNet-dipole have a physical basis, comparisons were made to charges computed using several population analysis techniques.

To perform the population analysis, DFT calculations were run using Gaussian09 [1]. The B3LYP [2, 3] functional and the cc-pVDZ basis set [4] were used. The population analyses that were performed are: CM5 [5], Mulliken [6], Hirshfeld [7] and Merz-Singh-Kollman (MSK) [8,9]. The molecular analogues were deemed fit as a references if the predicted charges corresponded to chemical intuition and the dipole moment was comparable to that calculated using DFT.

For the graphene sheet doped with nitrogen, a planar form of trimethylamine was used as a reference.

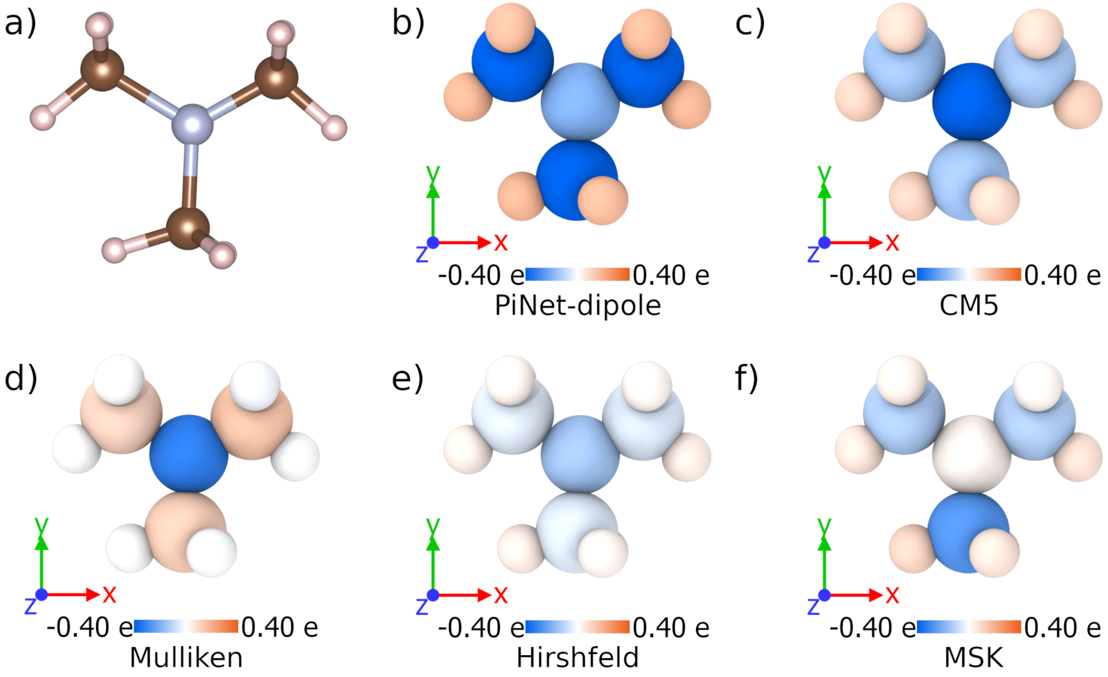

Figure S2: **Charges of trimethylamine**. The structure of trimethylamine (a). Computed with b) PiNet-dipole, c) CM5, d) Mulliken, e) Hirshfeld, and f) MSK.

Method	D_x (D)	D_y (D)	D_z (D)
DFT	-0.340	-0.286	-0.010
PiNet-dipole	-0.435	-0.169	-0.016
CM5	-0.126	-0.123	-0.013
MSK	-0.320	-0.280	-0.009

Table S1: **Dipole moment of trimethylamine**.

Element	PiNet charge (e)
N	-0.19527
C	0.03033
C	0.08597
C	0.07899

Table S2: **Base charges from trimethylamine as implemented in the N-doped graphene**.

The charges of the methyl groups are placed the carbon atoms when charges are used in the real system, to ensure a charge neutral entity.

For the graphene sheet doped with epoxy groups, ethylene oxide was used as the reference molecule.

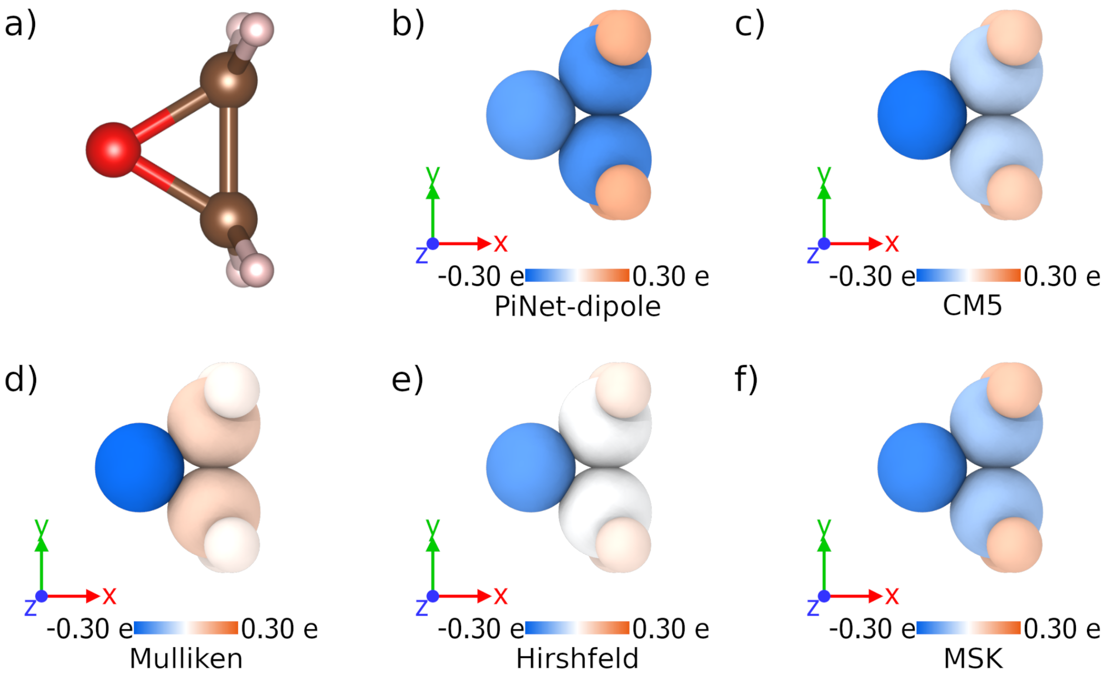

Figure S3: **Charges of ethylene oxide**. The structure of ethylene oxide (a). Computed with b) PiNet-dipole, c) CM5, d) Mulliken, e) Hirshfeld, and f) MSK.

Method	D_x (D)	D_y (D)	D_z (D)
DFT	1.806	0.000	-0.141
PiNet-dipole	1.811	0.000	-0.142
CM5	2.052	0.000	-0.161
MSK	1.837	0.000	-0.144

Table S3: **Dipole moment of ethylene oxide**.

Element	PiNet charge (e)
O	-0.19297
C	0.09648
C	0.09648

Table S4: **Based charges from ethylene oxide as implemented in the epoxy-terminated graphene oxide**.

Here, the charges of the hydrogen atoms are also combined with that of the carbon atoms when the charges are transferred to functionalized graphene. Once again, to ensure charge neutrality and to localize the charges on the graphene sheet.

For the graphene sheet doped with hydroxyl groups, methanol was used as the molecular analogue.

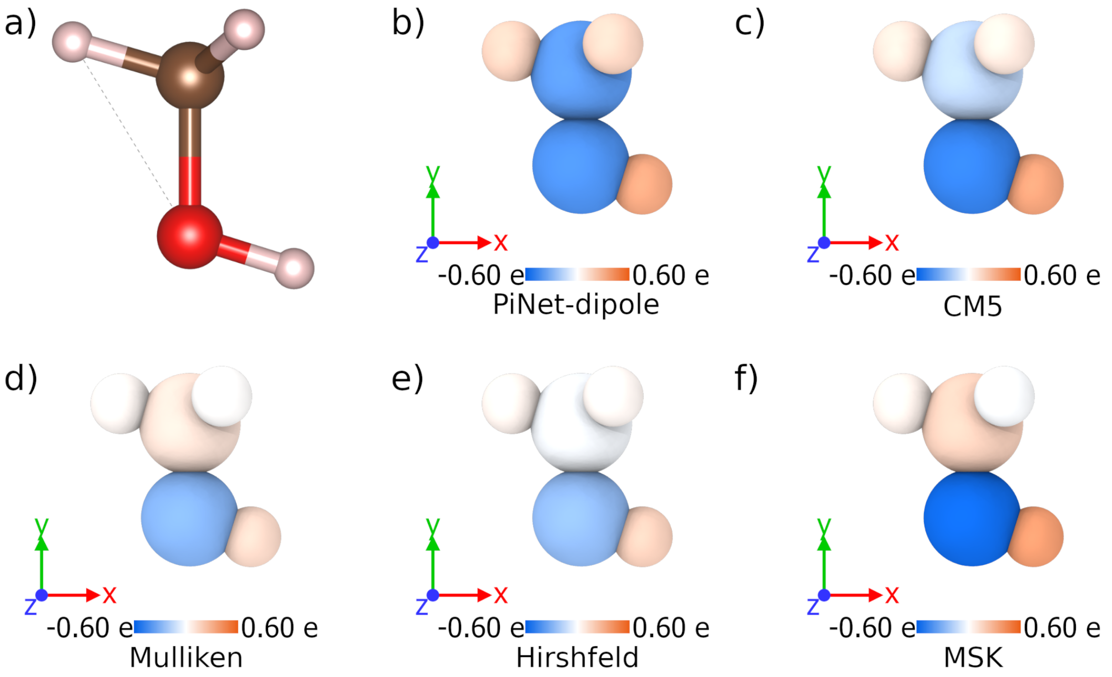

Figure S4: **Charges of methanol**. The structure of methanol (a). Computed with b) PiNet-dipole, c) CM5, d) Mulliken, e) Hirshfeld, and f) MSK.

Method	D_x (D)	D_y (D)	D_z (D)
DFT	1.340	0.825	0.000
PiNet-dipole	1.327	1.067	0.000
CM5	1.384	0.957	0.000
MSK	1.326	0.851	0.000

Table S5: **Dipole moment of methanol**.

Element	PiNet charge (e)
O	-0.41668
H	0.32197
C	0.09471

Table S6: **Base charges from methanol as implemented in the hydroxyl-terminated graphene oxide**.

The charge on the carbon atom is set so that it includes the charges of the hydrogen atoms as well. In this way, it compensates for the charge on the hydroxyl group, and the whole group is charge neutral.

Finally, for the graphene sheet functionalized with carboxyl groups, a smaller graphene flake with carboxyl groups was used as a reference. This was done because alternative analogues showed large fluctuations in the charges when changing the charge state of the analogue which did not correspond to chemical intuition. While the dipole moment for the carboxyl flake shows discrepancies to that of DFT in the x- and y-direction, the z-direction, which is the most important direction when it comes to the carboxyl group, agrees within a reasonable error margin.

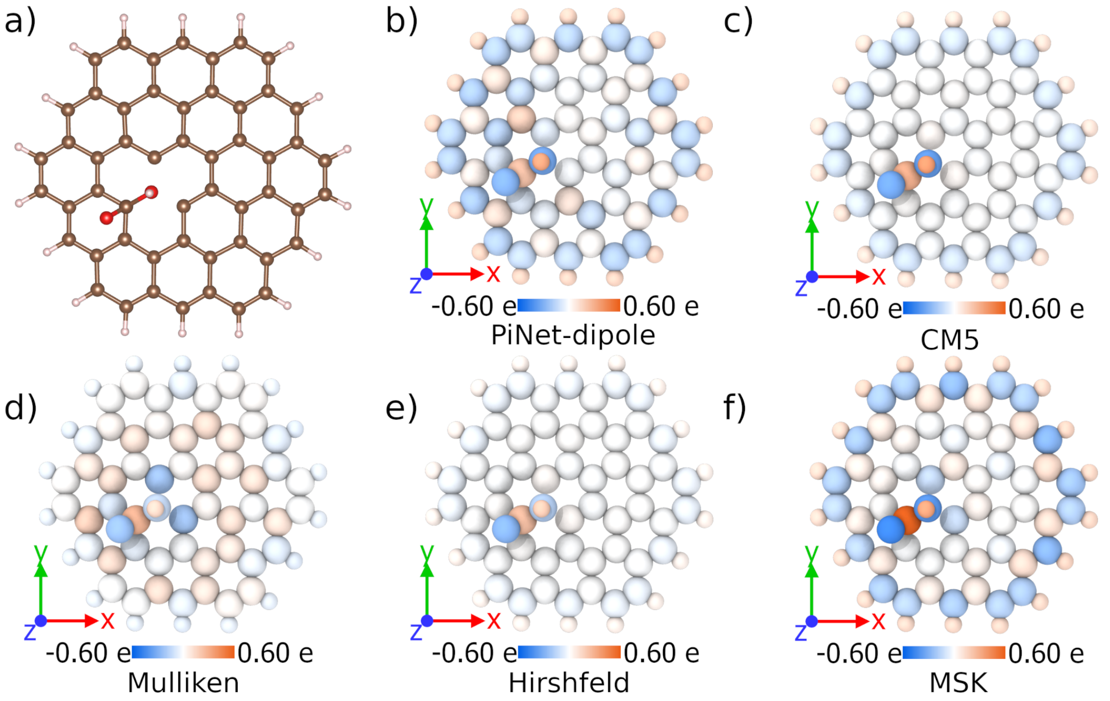

Figure S5: **Charges of the neutral carboxyl flake**. The structure of the neutral carboxyl flake (a). Computed with b) PiNet-dipole, c) CM5, d) Mulliken, e) Hirshfeld, and f) MSK.

Element	PiNet charge (e)
$O_{\text{double-bonded O}}$	-0.28245
O_{OH}	-0.31622
H_{OH}	0.32579
C	0.27288

Table S7: **Charges from the neutral carboxyl flake**.

Method	D_x (D)	D_y (D)	D_z (D)
DFT	0.2953	0.0873	0.3845
PiNet-dipole	3.301	1.699	0.145
CM5	0.435	0.230	0.297
MSK	0.265	0.087	0.354

Table S8: **Dipole moment from the neutral carboxyl flake**.

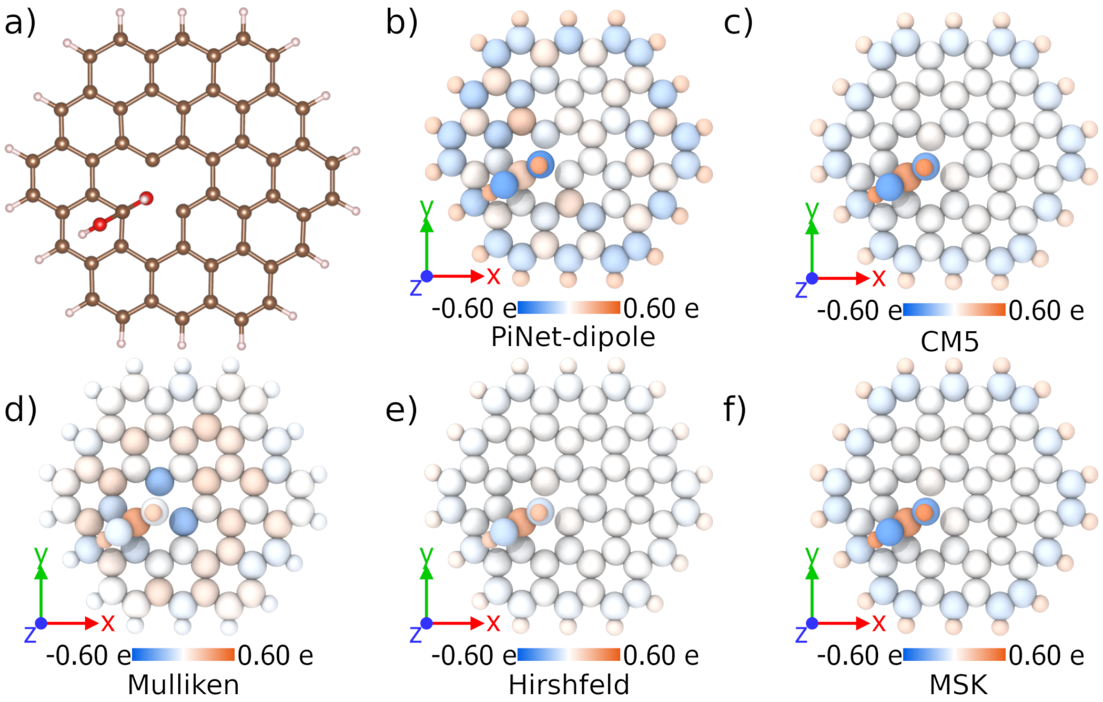

Figure S6: **Charges of the protonated carboxyl flake**. The structure of the protonated carboxyl flake (a). Computed with b) PiNet-dipole, c) CM5, d) Mulliken, e) Hirshfeld, and f) MSK.

Element	PiNet charge (e)
$O_{double-bonded\ O}$	-0.33339
$H_{double-bonded\ O}$	0.27653
O_{OH}	-0.36729
H_{OH}	0.33747
C	1.08668

Table S9: **Base charges from the protonated carboxyl flake as implemented in the protonated side of carboxyl-terminated graphene oxide**.

Since the investigated structures contain neutral, protonated, and deprotonated forms of the carboxyl groups, these are also the structures for which the charges were predicted. Here the charge of the carbon atom is set such that the total charge of the protonated carboxyl group is +1. Once again the charges of the atoms are predicted using PiNet-dipole. Then, the charge of the carbon atom is simply set to ensure that the charge of the protonated carboxyl group sums to +1. This is done to keep the charges localized, and because it is the simplest way to adjust the charge without the need for an arbitrary charge division. It also prevents unphysical modifications to the other charges from being made. This is supported by Figure S6, as this shows that the charge analysis performed with DFT methods the excess charge is also mostly located on the carbon atom.

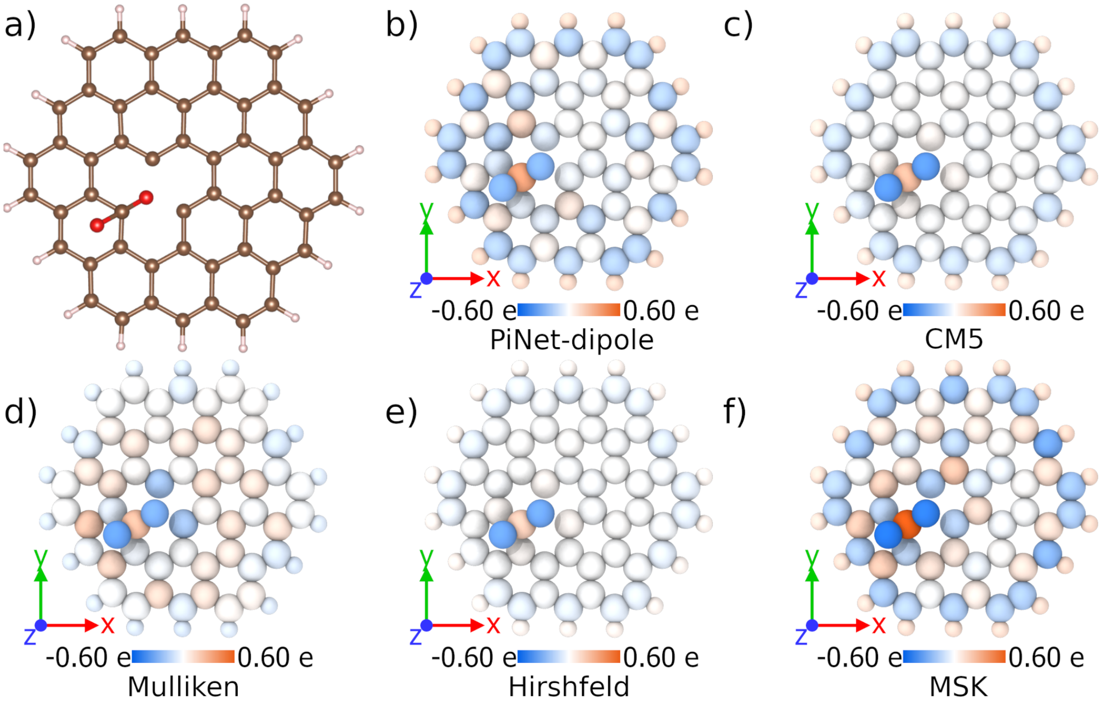

Figure S7: **Charges of the deprotonated carboxyl flake**. The structure of the deprotonated carboxyl flake (a). Computed with b) PiNet-dipole, c) CM5, d) Mulliken, e) Hirshfeld, and f) MSK.

Element	PiNet charge (e)
$O_{\mathrm{double-bonded\ O}}$	-0.28599
O_{OH}	-0.28723
C	-0.42678

Table S10: **Base charges from the deprotonated carboxyl flake as implemented in the deprotonated side of carboxyl-terminated graphene oxide**.

Similarly, for the deprotonated cases, the charge of the carbon atom is set such that the total charge of the deprotonated carboxyl group is -1. As can be seen in Figure S7, for the DFT charge methods the negative charge is spread across the carboyxl flake, mostly at the edges. As a first approximation, the excess charge is localized on the carbon atom in our implementation, which avoids any size-inconsistent charge divisions.

C Ion distributions at the electrified interfaces

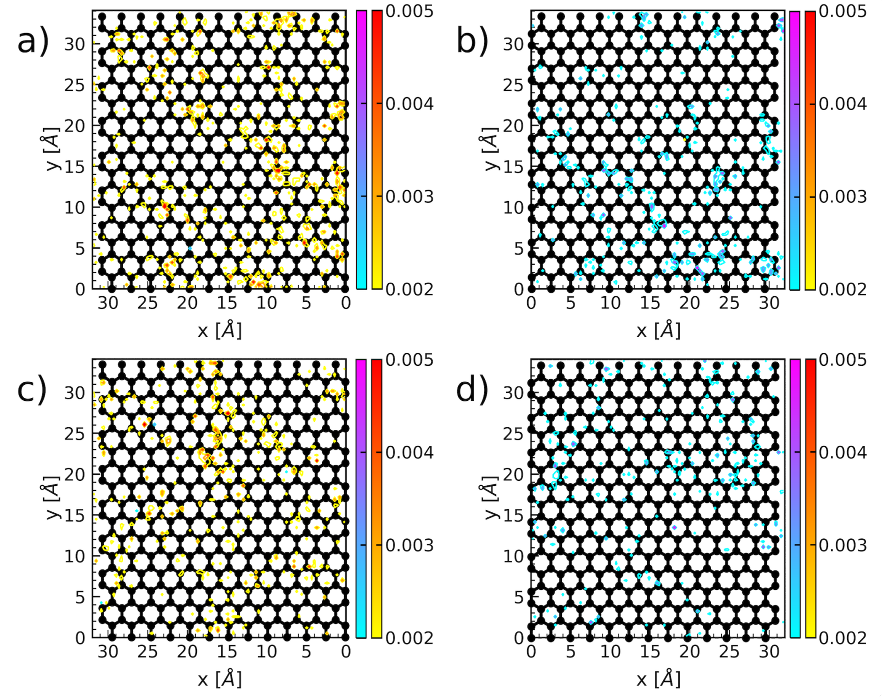

Figure S8: **Density of adsorbed ions in on a pristine graphene electrode** . Surface density in eÅ^{-2} of potassium (yellow to red color bar) and chloride (blue to purple color bar) using the MetalWalls (PM) on the negative (a) and positive (b) electrode, and using the PiNet-χ (EEM) model on the negative (c) and positive (d) electrode, under an applied potential of 2 V.

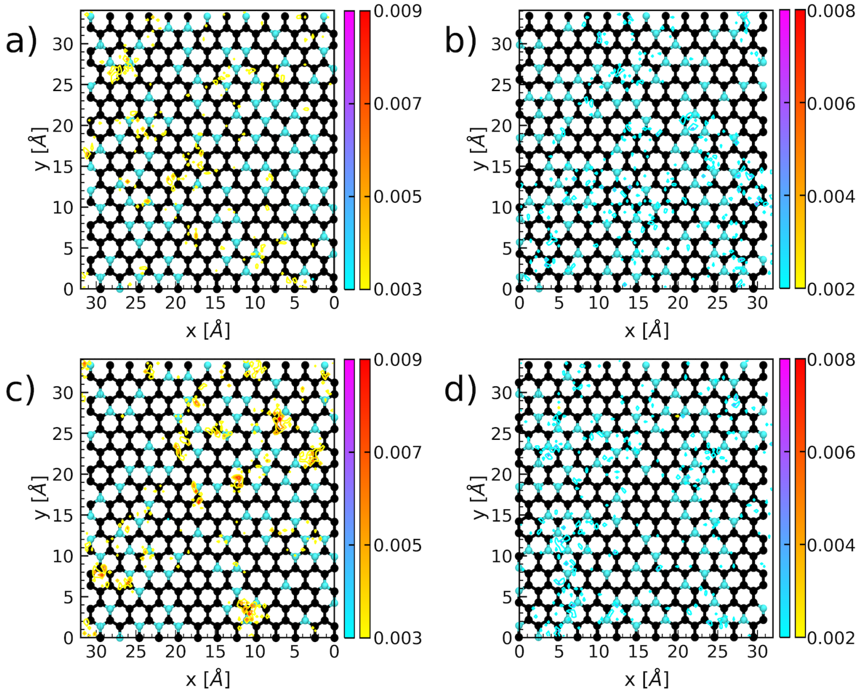

Figure S9: **Density of adsorbed ions on a graphene electrode with Nitrogen substitution**. Surface density in eÅ^{-2} of potassium (yellow to red color bar) and chloride (blue to purple color bar) using the MetalWalls (PM) model on the negative (a) and positive (b) electrode, and using the PiNet-χ (EEM) model on the negative (c) and positive (d) electrode, for a surface coverage of 20 % and under an applied potential of 2 V.

References

[1] M. J. Frisch, G. W. Trucks, H. B. Schlegel, G. E. Scuseria, M. A. Robb, J. R. Cheeseman, G. Scalmani, V. Barone, B. Mennucci, G. A. Petersson, H. Nakatsuji, M. Caricato, X. Li, H. P. Hratchian, A. F. Izmaylov, J. Bloino, G. Zheng, J. L. Sonnenberg, M. Hada, M. Ehara, K. Toyota, R. Fukuda, J. Hasegawa, M. Ishida, T. Nakajima, Y. Honda, O. Kitao, H. Nakai, T. Vreven, J. A. Montgomery Jr., J. E. Peralta, F. Ogliaro, M. Bearpark, J. J. Heyd, E. Brothers, K. N. Kudin, V. N. Staroverov, T. Keith, R. Kobayashi, J. Normand, K. Raghavachari, A. Rendell, J. C. Burant, S. S. Iyengar, J. Tomasi, M. Cossi, N. Rega, J. M. Millam, M. Klene, J. E. Knox, J. B. Cross, V. Bakken, C. Adamo, J. Jaramillo, R. Gomperts, R. E. Stratmann, A. J. Austin O. Yazyev, R. Cammi, C. Pomelli, J. W. Ochterski, R. L. Martin, K. Morokuma, V. G. Zakrzewski, G. A. Voth, P. Salvador, J. J. Dannenberg, S. Dapprich, A. D. Daniels, O. Farkas, J. B. Foresman, J. V. Ortiz, J. Cioslowski, and D. J. Fox, "Gaussian 09, Revision D.01 (Gaussian, Inc., Wallingford CT)" (2013).

[2] Axel D Becke, "Density-functional thermochemistry. I. The effect of the exchange-only gradient correction", J. Chem. Phys. **96**(3), pp. 2155–2160 (1992).

[3] Philip J Stephens, Frank J Devlin, Cary F Chabalowski, and Michael J Frisch, "Ab initio calculation of vibrational absorption and circular dichroism spectra using density functional force fields", J. Phys. Chem. **98**(45), pp. 11623–11627 (1994).

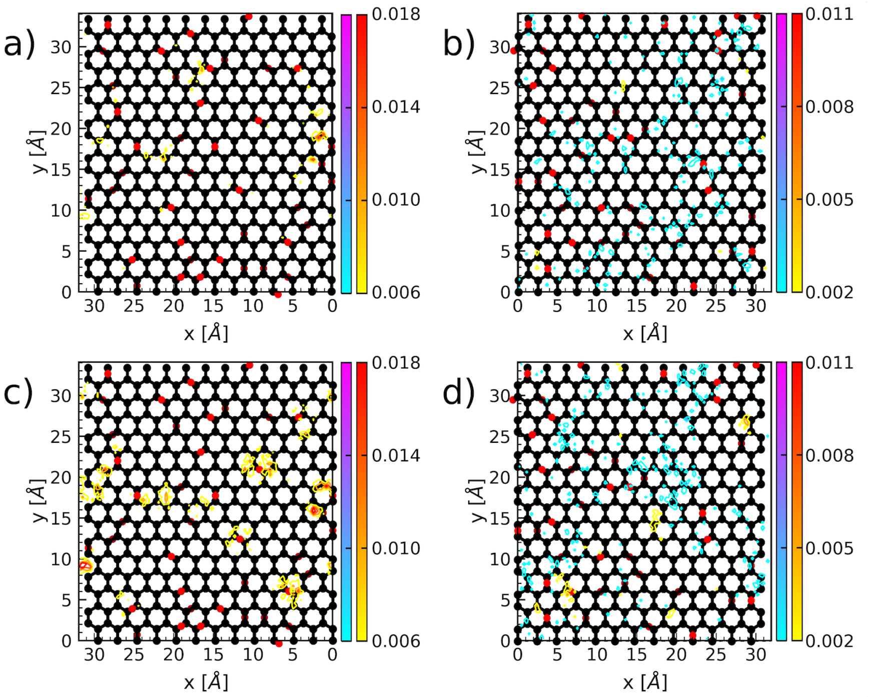

Figure S10: **Density of adsorbed ions on a graphene oxide electrode with epoxy terminations**. Surface density in e$Å^{-2}$ of potassium (yellow to red color bar) and chloride (blue to purple color bar) using the MetalWalls (PM) model on the negative (a) and positive (b) electrode, and using the PiNet-χ (EEM) model on the negative (c) and positive (d) electrode, for a surface coverage of 20 % and under an applied potential of 2 V.

[4] Thom H Dunning Jr., "Gaussian basis sets for use in correlated molecular calculations. I. The atoms boron through neon and hydrogen", J. Chem. Phys. **90**(2), pp. 1007–1023 (1989).

[5] Aleksandr V Marenich, Steven V Jerome, Christopher J Cramer, and Donald G Truhlar, "Charge model 5: An extension of Hirshfeld population analysis for the accurate description of molecular interactions in gaseous and condensed phases", J. Chem. Theory Comput. **8**(2), pp. 527–541 (2012).

[6] Robert S Mulliken, "Electronic population analysis on LCAO–MO molecular wave functions. I", J. Chem. Phys. **23**(10), pp. 1833–1840 (1955).

[7] Fred L Hirshfeld, "Bonded-atom fragments for describing molecular charge densities", Theor. Chim. Acta **44**(2), pp. 129–138 (1977).

[8] U Chandra Singh and Peter A Kollman, "An approach to computing electrostatic charges for molecules", J. Comput. Chem. **5**(2), pp. 129–145 (1984).

[9] Brent H Besler, Kenneth M Merz Jr, and Peter A Kollman, "Atomic charges derived from semiempirical methods", J. Comput. Chem. **11**(4), pp. 431–439 (1990).

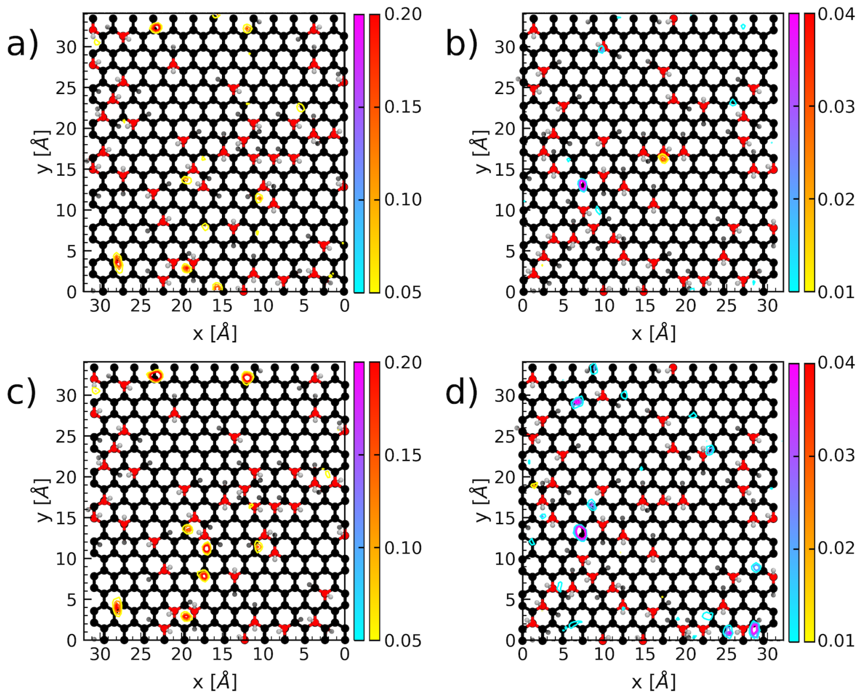

Figure S11: **Density of adsorbed ions on a graphene oxide electrode with hydroxyl terminations**. Surface density in eÅ^{-2} of potassium (yellow to red color bar) and chloride (blue to purple color bar) using the MetalWalls (PM) model on the negative (a) and positive (b) electrode, and using the PiNet-χ (EEM) model on the negative (c) and positive (d) electrode, for a surface coverage of 20 % and under an applied potential of 2 V.
